# Pre-surgical neoadjuvant oncolytic virotherapy confers protection against rechallenge in a murine model of breast cancer

**DOI:** 10.1038/s41598-018-38385-7

**Published:** 2019-02-12

**Authors:** Nikolas Tim Martin, Dominic Guy Roy, Samuel Tekeste Workenhe, Diana J. M. van den Wollenberg, Rob C. Hoeben, Karen Louise Mossman, John Cameron Bell, Marie-Claude Bourgeois-Daigneault

**Affiliations:** 1 Ottawa Hospital Research Institute, Centre for Innovative Cancer Research, Ottawa, K1H 8L6 Canada; 2 University of Ottawa, Department of Biochemistry, Microbiology and Immunology, Ottawa, K1H 8M5 Canada; 3 McMaster University, Department of Pathology and Molecular Medicine, Hamilton, ON Canada; 4 Department of Cell and Chemical Biology, Leiden University Medical Center, Leiden, The Netherlands; 5 CRCHUM – “Centre de recherche du Centre Hospitalier de l’Université de Montréal” and “Institut du cancer de Montréal”, Montreal, H2X 0A9 Canada; 6 “Département de microbiologie, infectiologie et immunologie, Faculté de Médecine, Université de Montréal”, Montreal, H3C3J7 Canada

## Abstract

The use of oncolytic viruses (OVs) for cancer treatment is emerging as a successful strategy that combines the direct, targeted killing of the cancer with the induction of a long-lasting anti-tumor immune response. Using multiple aggressive murine models of triple-negative breast cancer, we have recently demonstrated that the early administration of oncolytic Maraba virus (MRB) prior to surgical resection of the primary tumor is sufficient to minimize the metastatic burden, protect against tumor rechallenge, cure a fraction of the mice and sensitize refractory tumors to immune checkpoint blockade without the need for further treatment. Here, we apply our surgical model to other OVs: Vesicular stomatitis virus (VSV), Adenovirus (Ad), Reovirus (Reo) and Herpes simplex virus (HSV) and show that all of the tested OVs could positively change the outcome of the treated animals. The growth of the primary and secondary tumors was differently affected by the various OVs and most of the viruses conferred survival benefits in this neoadjuvant setting despite the absence of direct treatment following rechallenge. This study establishes that OV-therapy confers long-term protection when administered in the pre-operative window of opportunity.

## Introduction

While OVs were long believed to mediate their therapeutic activity through the direct killing of the cancer cells, it is now well accepted that the virus-triggered immune activation is a major factor contributing to the success of OV treatment. It is believed that oncolysis in the context of the stimulation of pattern recognition receptors by viral pathogen-associated molecular patterns induces anti-tumor immunity^1^. Furthermore, various studies have established that OV-therapy induces immunogenic cell death^2^, which is known to also trigger anti-tumor immunity. This has been demonstrated for Ad^3^, Measles virus^4^, Coxsackievirus^5^ and HSV^6^. Also, the inflammation caused by the infection contained within the tumor micro-environment has been shown by us and others to induce the production of chemokines that recruit immune cells to the tumor^7–9^. The clinical significance of this virus-induced inflammation is supported by two different in-human studies where the regression of distant lesions following local OV-therapy was reported (Vaccinia virus (VV) for hepatocellular carcinoma^10^ and HSV for melanoma^11^ treatment). Importantly, these clinical findings are in line with preclinical work conducted using murine models of cancer, which demonstrated that various OVs (MRB^8^, Reo^12^, Newcastle disease virus^13^, VV^14^, Measles virus^15^ and HSV^16^) could sensitize refractory tumors to immune checkpoint blockade and control not only the treated tumors, but also natural metastases. This important finding further established the therapeutic importance of OV-therapy-induced anti-tumor immunity as well as the long-term benefits provided by the treatment.

In a recent study, we demonstrated that pre-operative OV-therapy of breast cancer using MRB was sufficient to achieve a complete response in a fraction of the mice treated and protect against rechallenge without the need for additional treatment^8^. This previous study was a proof of concept for the use of MRB for breast cancer treatment in the window of opportunity that separates the diagnosis and surgery but whether other OVs also act similarly remains to be determined. Here, we tested 4 additional OVs: VSV, Ad, Reo and HSV and found that all but Reo could efficiently confer protection against tumor rechallenges and improve survival from 5 to 10 days. Furthermore, our results show that MRB, VSV, Ad and HSV could all efficiently control the growth of secondary tumors and cure 10 to 20% of the mice. Our results establish the long-term benefits of using different OVs to treat cancer before surgery.

## Results

### Most OVs control primary tumors

**Figure 1:**
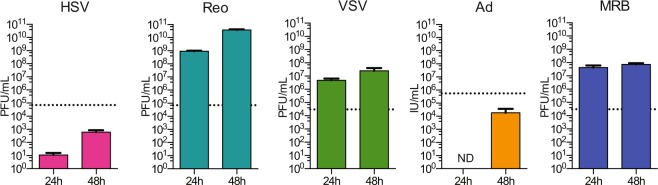
Replication of the different OVs in the 4T1 cell line. Virus outputs obtained from monolayers of 4T1 cells infected with HSV, Reo, VSV, Ad and MRB for 24 and 48 h. The dashed lines represent the respective amount of each virus used for infection.

**Figure 2:**
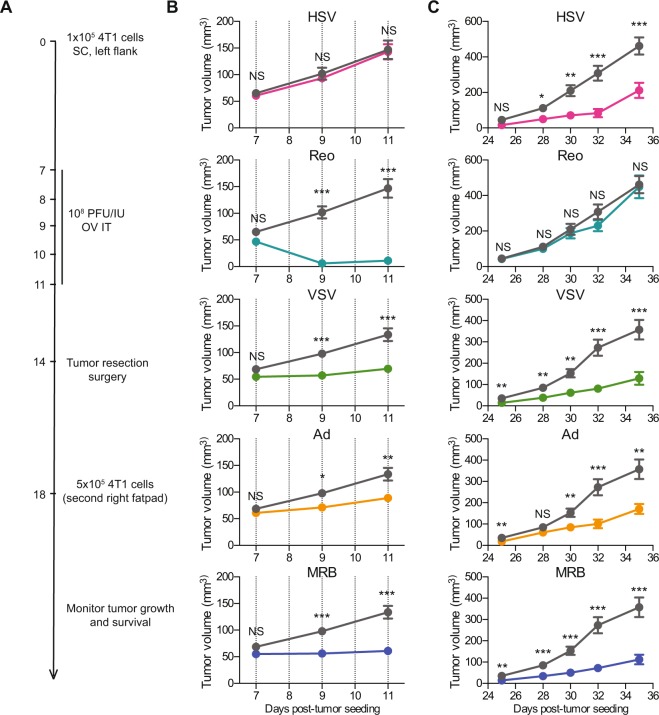
Various OVs differentially control primary and secondary tumor growth. (**A**) Treatment schedule used in the tumor-rechallenge model. (**B**) Growth curves of primary tumors directly treated with the different OVs or untreated. (**C**) Growth curves of secondary tumors. The dashed lines represent the days of treatment and the error bars represent the standard error of the mean, (n = 10 per group). NS: p > 0.05, *p < 0.05, **p < 0.01, ***p < 0.001 (multiple unpaired t-test).

Before testing the efficacy of the different OVs *in vivo*, we first assessed the replication of the different viruses *in vitro*. We used the 4T1 triple-negative breast cancer model since we have already established that early treatment with MRB virus confers protection against rechallenge using this cell line^8^. We infected monolayers of 4T1 cells with the different viruses at a multiplicity of infection of 0.1 and quantified the viral output 24 and 48 h later. Our results show that VSV, Reo and MRB could all replicate in the tumor cell line while the virus amounts recovered from the Ad and HSV conditions were lower than their respective inputs (Fig. 1). In order to determine if different OVs had the capacity to confer protection against secondary tumors when only treating the primary tumors, we took advantage of our tumor rechallenge model, depicted in Fig. 2A. HSV, Reo, VSV, Ad and MRB were all injected intratumorally (IT) for five consecutive days when the tumors reached an average volume of approximately 50 mm^3^. Our results show that Reo, VSV, Ad and MRB could all efficiently slow tumor growth (n = 10–20) (Fig. 2B and Supplementary Fig. 1). Notably, Reo was extremely efficient in this model and completely eradicated some of the tumors. On the other hand, HSV did not affect the growth of the injected tumors.

### Most OVs protect against rechallenge and improve survival when administered prior to surgery

**Figure 3:**
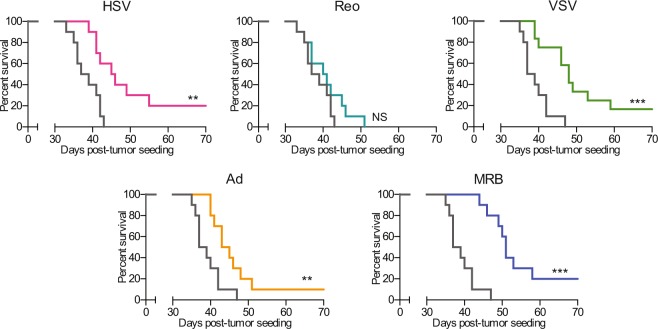
Most OVs confer therapeutic benefits when administered prior to surgery. Kaplan-Meier survival analysis of the animals treated with the different OVs using the tumor rechallenge model (n = 10 per group). NS: p > 0.05, **p < 0.01, ***p < 0.001 (Mantel-Cox test).

When looking at the secondary 4T1 tumors (implanted after the primary tumors were surgically removed), we found that HSV, VSV, Ad and MRB could all confer protection in this rechallenge setting with the animals from these groups showing smaller tumors compared to untreated animals (n = 20) (Fig. 2C and Supplementary Fig. 1). This time, Reo did not confer any protection to the treated animals. Notably, the 4 OVs that protected against the tumor rechallenge could all provide survival benefits with an improvement of the median survival ranging from 5 to 10 days and 10 to 20% of the animals being cured and completely refractory to the secondary tumors (Fig. 3). Reo, which failed at protecting the mice against rechallenge, did not significantly improve the survival of the animals compared to the untreated control group.

### OV-therapy changes the outcome of the treated animals

**Figure 4:**
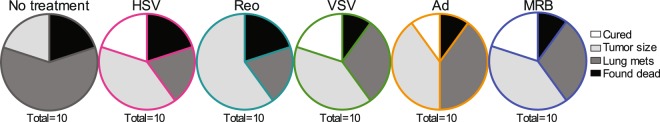
Neoadjuvant OV-therapy improves the outcome of mice. Cause of death of 4T1 tumor-bearing mice in the tumor-rechallenge model.

Over the course of the experiments, we noticed that the overall condition of OV-treated mice from the different groups deteriorated and the animals reached end-point for different reasons compared to the untreated ones. We monitored the mice, recorded their outcome and classified each animal into one of the four following categories: cured, sacrificed because of the tumor size (reached humane endpoint), lung tumors (the animals were in severe respiratory distress) and found dead. Interestingly, we observed that all of the OVs tested favorably changed the outcome of the mice (Fig. 4). Not only could most viruses cure some of the mice, but also a bigger fraction of the animals was end-point because of the local growth of the secondary tumors instead of the dissemination of the cancer to the lungs, as was seen most often in the untreated control group.

## Discussion

Our study establishes the long-term benefits provided by early OV-therapy. Using our tumor rechallenge model, we observed 3 different types of responses: (I) the OV efficiently controlled the growth of the primary tumor and conferred therapeutic benefits (Reo); (II) the OV positively changed the outcome of the mice and efficiently controlled the secondary tumor only (HSV); (III) OV treatment before surgery provided therapeutic benefits and controlled tumor growth of the primary as well as the rechallenge tumor (MRB, VSV and Ad). The head-to-head comparison of the different viruses using the same experimental conditions allowed us to evaluate the therapeutic activity of each OV, but it is important to consider that this strategy came to the disadvantage of some viruses and therefore could have led to the under-estimation of the efficacy of some OVs. For example, the production of HSV has been found to peak 72 h post-infection in 4T1 cells^17^ and the surgeries were performed 72 h after the last viral treatment in our model, which may have prevented the virus from achieving maximal oncolysis in our experimental model. In line with this, we did not detect HSV replication above input in our 4T1 cells line at 24 h and 48 h post-infection. On the other hand, the production of VSV, MRB and Reo in this same cell line is already significant after 24 h, which favors these OVs in our model. Also, as shown by our data, the Ad5 virus used, which harbors a deletion of the E1A and E1B regions, does not replicate in the 4T1 tumor cells, which underlies its limited oncolytic activity in the primary tumor. Nevertheless, early Ad and HSV treatments still affected the growth of the secondary tumors. This can be attributed to an adjuvant function of even replication-incompetent adenoviruses^18^. Interestingly, although Reo treatment provided the best response in the treated tumors and changed the outcome of the mice, it did not affect the size of the secondary tumors or the overall survival of the animals: no cures were observed but a smaller fraction of the mice succumbed to systemic spread of the disease. The almost complete eradication of primary tumors by Reo potentially minimized the early metastasis of the tumors, which could explain why fewer animals from this group succumbed to lung metastases.

Importantly, our results using HSV demonstrated that the virus failed at controlling primary tumor growth within 4 days, but still prolonged survival and reduced the fraction of the animals succumbing to lung metastases. These results are in line with a study published by another group using the 4T1 cell line in a similar treatment model that showed no significant difference in tumor size before 6–8 days post-injection and also that the virus could efficiently limit the lung metastases^17^. Given that we removed the primary tumors soon after treatment, we cannot determine if HSV would have been able to control the treated tumor at later time points, but we can still conclude that the HSV treatment before surgery provides therapeutic benefits. Finally, the best results obtained in our tumor rechallenge model were using VSV, Ad and MRB, which could all control the growth of both tumors and cure some of the animals. Our results showing that the oncolytic virus-treated animals are protected against a tumor rechallenge are in line with the induction of anti-tumor immunity by oncolytic virotherapy, which has been shown in previous studies by us and others^7,8,19^. Taken together, our data support our previous proof of concept study and demonstrate that different OVs can be administered before surgery to confer protection against relapse and limit metastasis.

## Methods

### Cell lines and culture

The 4T1, Vero and 293X-adeno cell lines were obtained from ATCC and maintained in Dulbecco’s Modified Eagle’s Medium (DMEM) (Corning Cellgro) supplemented with 10% fetal bovine serum (Sigma Life Science) and cultured at 37 °C with 5% CO_2_.

### Viruses, production and quantification

All experimental protocols were approved by the University of Ottawa biosafety committee. The MRB virus used in this study is the oncolytic variant MG1^20^. The VSV used in this study is the attenuated mutant Δ51 of the Indiana strain^21^. VSV and MRB were expanded on Vero cells, purified by centrifugation and quantified by plaque assay as described previously^7^. The Ad used in this study is a E1A/E1B-deleted mutant of Ad5. Ad was amplified on 293X-adeno cells, purified by ultracentrifugation on caesium chloride density gradient (as described previously^22^) and quantified using the Adeno-X™ Rapid Titer Kit (Clontech) as per the manufacturer’s protocol. The Reo used in this study was the mammalian orthoreovirus T3D strain R124 that was amplified on HER911 cells, purified by caesium chloride centrifugation, and titrated by plaque assay, as described previously^23^. The oncolytic HSV-1 used in this study has a deletion of the *ICP0* coding region and has been previously described^6^. HSV-1 was propagated and tittered on U2OS cells in the presence of 3 mM hexamethylene bisacetamide (Sigma). HSV-1 purification and concentration was done using sucrose cushion ultracentrifugation^6^.

### Tumor rechallenges model of neo-adjuvant treatment

All experiments were performed in accordance with the University of Ottawa ACVS guidelines. 4T1 tumor cells (10^5^) were injected in the left flank of 6–8 weeks old Balb/C mice (Charles Rivers laboratories). 7 days post-tumor seeding, palpable tumors were treated daily with intratumoral injections of the different viruses (all at doses of 10^8^ plaque forming units (PFU) or infectious units (IU)) from day 7 to day 11, for a total of 5 injections. The tumors we surgically removed on day 14 and the animals were rechallenged by the injection of a higher dose of 4T1 cells (5 × 10^5^) in the second right mammary fatpad on day 18. The take rates of both the primary and secondary tumors are 100% using these doses of tumor cells and all the animals survived the surgical procedure. The wellness of the animals was assessed daily and tumor growth was monitored using a digital caliper. Description of endpoints: as per institutional guidelines, the animals were sacrificed when their tumor size reached 1500 mm^3^, the outcome of these animals was classified as: death because of the tumor size. The animals that showed signs of respiratory distress were euthanized and the lungs were inspected for the presence of lung tumors. Lung metastases were found in all of these animals and they were labelled as: dead because of lung mets. A fraction of the animals were found dead. The conditions in which some of the bodies were found did not allow for the determination of the cause of death by necropsy. Therefore, these animals were simply classified as: found dead. Finally, the animals that were still alive 100 days post-tumor challenge were sacrificed and examined for the presence of tumors at both injection sites as well as in the lungs. All of these mice were free of visible tumors at this time point and were labelled as: cured.

### Statistical analysis

The statistical analyses were performed using GraphPad Prism 6.0 as described in the figure legends.

## Supplementary information

Supplementary Dataset 1
