# Supplementary material for: Pre-surgical neoadjuvant oncolytic virotherapy confers protection against rechallenge in a murine model of breast cancer
Source: Sci Rep. 2019 Feb 12;9:1865. doi: 10.1038/s41598-018-38385-7 (PMC6372691; doi:10.1038/s41598-018-38385-7)
Supplement: Supplementary file 1 — Supplementary Dataset 1 [file 41598_2018_38385_MOESM1_ESM.docx]

**Pre-surgical neoadjuvant oncolytic virotherapy confers protection against rechallenge in a murine model of breast cancer**

Nikolas Tim Martin^1,2*^, Dominic Guy Roy^1,2*^, Samuel Workenhe^3^, Diana J.M. van den Wollenberg^4^, Rob C. Hoeben^4^, Karen Mossman^3^, John Cameron Bell^1,2^ and Marie-Claude Bourgeois-Daigneault^5,6^.

^1^ Ottawa Hospital Research Institute, Centre for Innovative Cancer Research, Ottawa, K1H 8L6, Canada.

^2^ University of Ottawa, Department of Biochemistry, Microbiology and Immunology, Ottawa, K1H 8M5, Canada.

^3^ McMaster University, Department of Pathology and Molecular Medicine, Hamilton, ON, Canada.

^4^ Department of Cell and Chemical Biology, Leiden University Medical Center, Leiden, The Netherlands.

^5^ CRCHUM – “Centre Hospitalier de l’Université de Montréal” and “Institut du cancer de Montréal”, Montreal, H2X 0A9 Canada.

^6^ “Département de Microbiologie, d’Infectiologie et d’Immunologie, Faculté de Médecine, Université de Montréal”, Montreal, H3C3J7, Canada

^*^ These authors contributed equally

**Supplementary Table 1:** Data collected from each mouse used in the study.
